# Wearable cardioverter-defibrillators after myocardial infarction: a review of its clinical utility and unmet needs in current clinical practice

**DOI:** 10.1007/s12928-021-00788-1

**Published:** 2021-07-01

**Authors:** Hirofumi Hioki, Ken Kozuma, Yoshio Kobayashi, Kenji Ando, Yoshihiro Morino, Jun Kishihara, Junya Ako, Yuji Ikari

**Affiliations:** 1 Division of Cardiology, Teikyo University School of Medicine, 2-11-1 Kaga, Itabashi-ku, Tokyo, 173-8605 Japan; 2 Department of Cardiovascular Medicine, Chiba University Graduate School of Medicine, Chiba, Japan; 3 Department of Cardiology, Kokura Memorial Hospital, Kitakyushu, Japan; 4 Department of Cardiology, Iwate Medical University Hospital, Morioka, Japan; 5 Department of Cardiovascular Medicine, Kitasato University School of Medicine, Sagamihara, Japan; 6 Department of Cardiovascular Medicine, Tokai University School of Medicine, Isehara, Japan

**Keywords:** Sudden cardiac death, Myocardial infarction, Wearable cardioverter-defibrillator, Left ventricular dysfunction, Prognosis

## Abstract

Sudden cardiac death is one of the leading causes of death in the older population. Compared with the general population, patients who experienced a myocardial infarction are four to six times more likely to experience sudden cardiac death. Though primary percutaneous coronary intervention considerably reduces mortality in patients who experienced a myocardial infarction, a non-negligible number of sudden cardiac deaths still occurs. Despite the high incidence rate of sudden cardiac deaths during the first month after myocardial infarction, prophylactic use of implantable cardioverter-defibrillators has so far failed to convey a survival benefit. Therefore, current clinical guidelines recommend that cardioverter-defibrillator implantation is contraindicated until 90 days after myocardial infarction. Wearable cardioverter-defibrillators were first approved for clinical use in 2002 and are currently considered as a bridge to therapy in patients with myocardial infarction with a reduced left ventricular ejection fraction in whom cardioverter-defibrillator implantation is temporarily not indicated. However, there is insufficient recognition among interventional cardiologists of the use of wearable cardioverter-defibrillators for preventing sudden cardiac death after myocardial infarction. Hence, we reviewed the evidence of the efficacy of wearable cardioverter-defibrillators used in patients following myocardial infarction to achieve better management of sudden cardiac death.

## Introduction

Sudden cardiac death (SCD) is a fatal complication in patients who have experienced a myocardial infarction MI [1]. In fact, epidemiologic studies have demonstrated that an estimated four to five million people die from SCD annually [2]. Among patients who experience MI, a low left ventricular ejection fraction (LVEF) is strongly associated with SCD soon thereafter [3]. One randomized clinical trial indicated that implantable cardioverter-defibrillators (ICDs) reduce the risk of SCD fourfold in patients who experience MI and LVEF when implantation is performed months to years after MI [4]. Later randomized controlled trials did not provide empirical support for ICD implantation during the early phases, particularly within 40 days, after MI, because of an increased risk of non-arrhythmic death [5–7]. In cases where ICD implantation is not indicated, wearable cardioverter-defibrillators (WCDs) can serve as a bridge to definitive therapy.

WCDs have been approved for clinical application in Japan since 2014 and are used in selected patients at high risk for SCD. Notably, WCDs have many advantages compared to conventional ICDs, including the non-invasiveness of the procedure and the ease with which it can be terminated as soon as it is considered unnecessary. Despite its feasibility and clinical utility, WCDs are little known among interventional cardiologists. Therefore, we reviewed the indications for and studies of WCDs in patients who experienced an MI and are at high risk for SCD.

## Prevalence of SCD after MI

**Fig. 1:**
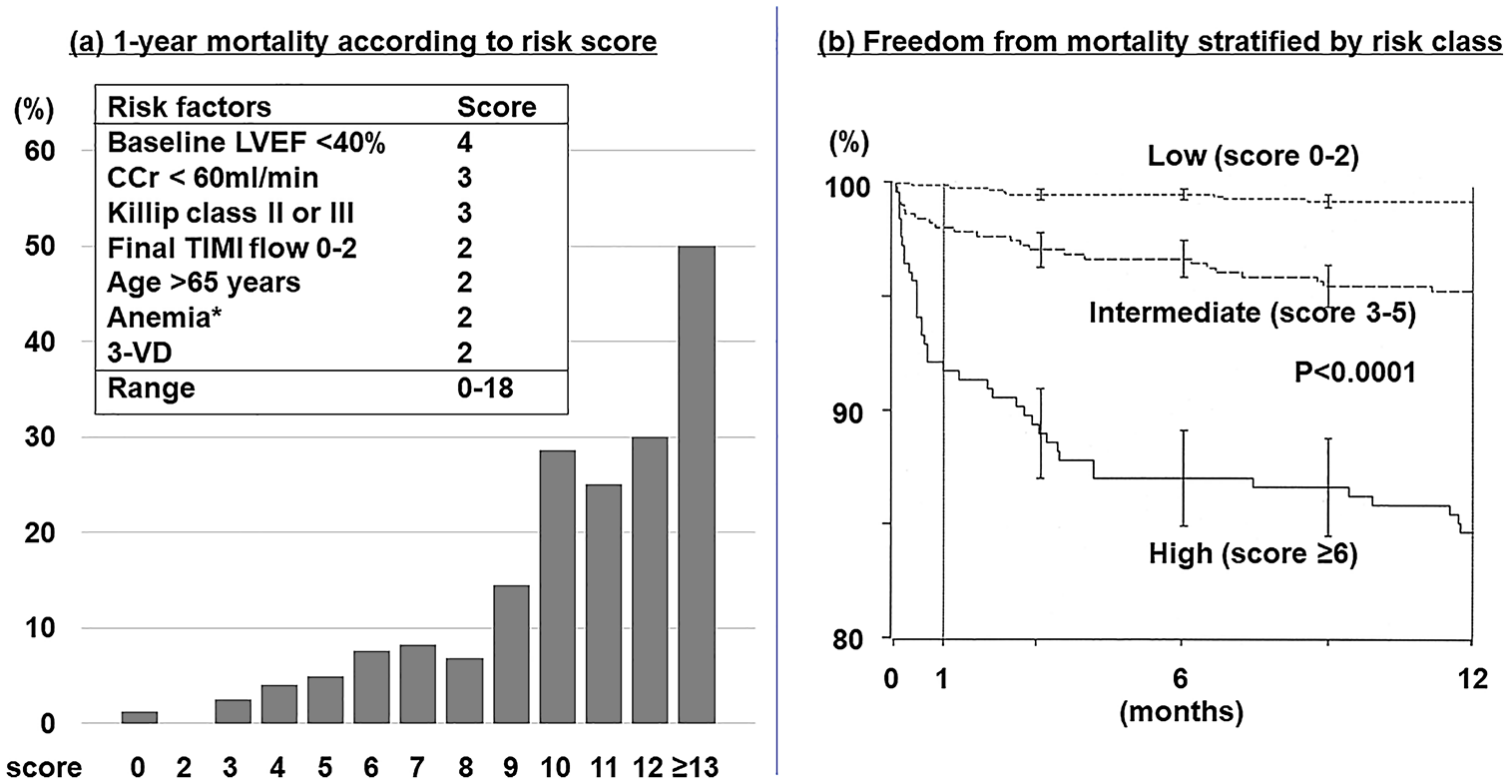
CADILLAC risk score and mortality. The incidence of mortality increased steeply at a risk score of ≥ 10 (**a**). Three strata of risk score, defined as low, intermediate, and high, demonstrated prognostic utility throughout the 1-year follow-up (**b**)

The previous Japanese registry data showed that acute-phase ventricular arrhythmia, which is a cause of SCD, was observed in one-fifth of MI patients and associated with higher incidence of in-hospital mortality [8]. From the results of observational and randomized controlled studies, the incidence rate of SCD after MI is 2–4% per year in Western countries [3, 9]. From the pooled data of two cohort studies in Japan, the incidence of SCD after MI is 1.2% [10]. Importantly, the risk of SCD after MI is time-dependent, with a tenfold higher risk in the first 30 days after MI, particularly in patients with an impaired LVEF; the risk decreases exponentially over the first 6 months and plateaus after 12 months [3]. Although a combination of early revascularization and medication, including beta-blockers, statins, anti-platelet therapy, angiotensin-converting enzyme inhibitors/angiotensin receptor blockers, and mineralocorticoid receptor antagonists reduces the mortality risk in the early phases after MI, SCD remains a non-negligible cause of death [11–13]. A low LVEF after MI, particularly ≤ 30%, is a powerful independent predictor of SCD, and the mortality risk in patients with a low LVEF after MI increases as a function of elapsed time from the MI [14, 15]. In the primary percutaneous coronary intervention (PCI) era, Halkin et al. [16] reported a risk score for predicting mortality after MI, which they developed from the Controlled Abciximab and Device Investigation to Lower Late Angioplasty Complications (CADILLAC) trial and validated using data from the Stent-Primary Angioplasty in Myocardial Infarction trial. This scoring system is comprised of clinical and angiographic parameters and is useful for predicting short- and long-term mortality, including from SCD, in patients who experienced MI (Fig. 1). As a number of patients are at risk of SCD after MI, their optimal management is warranted. Although, in the late post-MI period, ICD implantation yielded a survival benefit in patients with a low LVEF, ICD implantation for primary prevention of SCD in the early phases after MI failed to reduce overall mortality due to an increase in non-SCD and other adverse events, including infection, inappropriate shock, and heart failure [4–7]. Of note in those studies is the fact that a number of patients regained full cardiac function after the early phases after MI. Indeed, a recent prospective study demonstrated that, in patients with a LVEF ≤ 35% following MI, 57% of patients experienced an improvement in LVEF to > 35% within 3 months of the MI [17]. In other words, nearly 60% of patients with a LVEF < 35% during the acute phase of MI do not require ICD implantation. Therefore, an alternative to early ICD implantation is required during the first 40 days after MI in patients with a low LVEF.

## Overview of WCDs

**Fig. 2:**
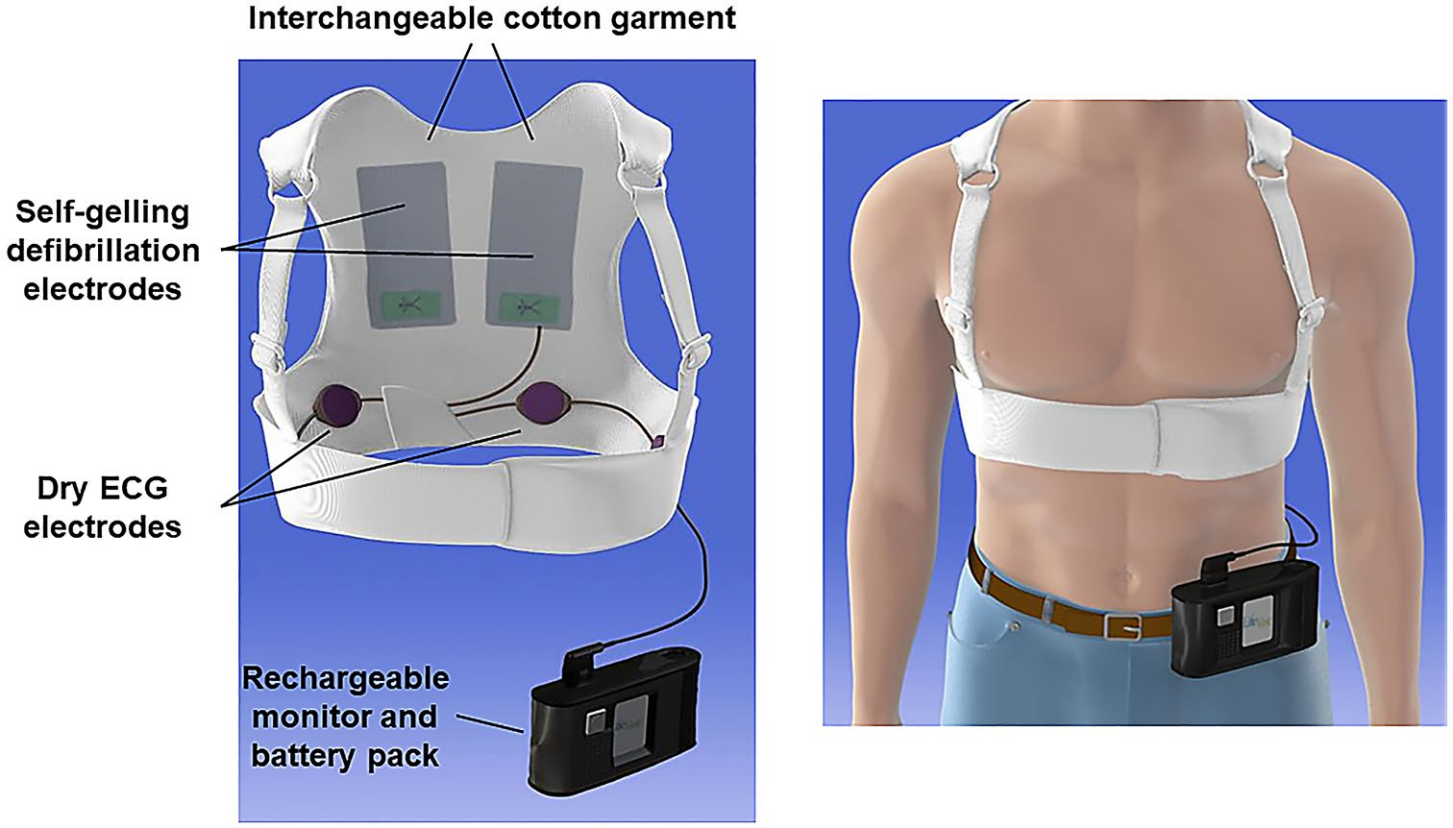
Wearable cardioverter-defibrillator

WCDs are a possible alternative to ICDs for preventing SCD in the early phases after an MI. The WCD (LifeVest, ZOLL Medical Corporation, Chelmsford, MA) is a unique medical tool, comprised of a detachable elastic vest with three non-adhesive defibrillation electrodes and four non-adhesive sensing electrocardiogram electrodes fitted to the chest, as well as a monitor unit at the waist (Fig. 2) [18]. Similar to the conventional ICD, the WCD uses an arrhythmia detection algorithm before delivering a shock; the algorithm is based on heart rate, template matching, and persistence of arrhythmia [19]. After arrhythmia detection, the WCD alerts the patient with vibrations, LED signals, and an audible warning alarm. This gives the patient time to cancel the impending shock by pressing the response button of the monitor unit. If the response button is not pressed, the WCD starts the therapy by extruding gel from electrodes inside the vest and defibrillates the patient’s heart. In spite of its simplicity, the device’s diagnostic sensitivity and specificity are reportedly comparable to those of ICDs [20–22]. To date, the efficacy of WCDs adapted to Japanese patients has not been established, although the Japan Wearable Cardioverter Defibrillator Registry (J-WCDR) is under way; it is a prospective observational registry aimed to evaluate “the actual state of WCD use in Japan and its clinical consequences” (UMIN000040881).

## Indication for WCD use in patients who experience MIs

As use of an ICD to prevent SCD during the first 30 days after an MI is not associated with a lower mortality rate, Japanese, American, and European guidelines state that ICD implantation should only be considered from one month after an MI occurred [3, 6, 7, 23]. In these guidelines and consensus documents, WCDs are recommended as a “bridging therapy” in patients at high risk for SCD during this 1-month period [24–26] (Table 1).

**Table 1:** Summary of guideline recommendations for wearable cardioverter-defibrillators

Class	2015 ESC guidelines	2016 Science advisory from the AHA	2017 AHA/ACC/HRS guideline	2018 JCS guideline
Class IIa	WCD should be considered for bridging until full recovery or ICD implantation inpatients after inflammatory heart diseases with residual severe LV dysfunction and/or ventricular electrical instability	Use of WCD is reasonable when there is a clear indication for an implanted/permanent device accompanied by a transient contraindication or interruption in ICD care such as infection		WCD should be considered in the following cases (1) Patients with LVEF ≤ 35% and symptoms of heart failure (NYHA class II–III) within 40 days from MI or within 90 days from revascularization (2) Acute decompensated heart failure with LVEF ≤ 35% due to non-ischemic cardiac disease within 90 days of its onset (3) Candidates of cardiac transplantation with irreversible end-stage heart failure (4) Clear indication for ICD implantation with temporal contraindication (e.g., infection) (5) Cases requiring temporary ICD extraction due to infection
Class IIb	(1) WCD may be considered for adult patients with poor LV systolic function who are at risk of sudden arrhythmic death for a limited period, but are not candidates for an ICD (arrhythmias in the early post-myocardial infarction phase) (2) ICD implantation or temporary use of a WCD may be considered within 40 days after MI in selected patients (incomplete revascularization, pre-existing LV dysfunction, occurrence of arrhythmia > 48 h after the onset of ACS, polymorphic VT or VF)	(1) Use of WCDs is reasonable as a bridge to more definitive therapy such as cardiac transplantation (2) Use of WCDs may be reasonable when there is concern about a heightened risk of SCD that may resolve over time or with treatment of LV dysfunction; for example, in ischemic heart disease with recent revascularization, newly diagnosed non-ischemic dilated cardiomyopathy in patients starting guideline-directed medical therapy, or secondary cardiomyopathy in which the underlying cause is potentially treatable (3) WCDs may be appropriate as bridging therapy in situations associated with an increased risk of death in which ICDs have been shown to reduce SCD but not overall survival, such as within 40 days of MI	In patients at an increased risk of SCD but who are not eligible for an ICD, e.g., having LVEF < 35%, within 40 days from MI, and/or revascularization within the past 90 days, the WCD may be reasonable	
Class III		WCDs should not be used when the non-arrhythmic risk is expected to significantly exceed the arrhythmic risk, particularly in patients who are not expected to survive > 6 months		

*ACC* American College of Cardiology, *ACS* acute coronary syndrome, *AHA* American Heart Association, *ESC* European Society of Cardiology, *HRS* Heart Rhythm Society, *ICD* implantable cardioverter-defibrillator, *JCS* Japanese Circulation Society, *LV* left ventricular, *LVEF* left ventricular ejection fraction, *MI* myocardial infarction, *NYHA* New York Heart Association, *SCD* sudden cardiac death, *VF* ventricular fibrillation, *VT* ventricular tachycardia, *WCD* wearable cardioverter-defibrillator

## Clinical efficacy of WCD

There have been several studies in which the clinical efficacy of WCDs has been examined (Table 2); the use of WCDs was discovered to reduce mortality in selected patients at high risk for SCD following an MI. The WEARIT and BIROAD studies were retrospective studies of patients with symptomatic heart failure and a LVEF < 30% (WEARIT cohort) or those at high risk for SCD after an MI or bypass surgery (BIROAD cohort). In those studies, 289 patients were enrolled and used WCDs, with a 75% successful defibrillation rate [22]. In a prospective analysis of a large U.S. national registry, patients with a LVEF < 35% had a higher rate of early mortality after PCI compared to late mortality; moreover, WCD use was associated with a lower risk of both early and long-term mortality [27]. The WEARIT-II registry was the first prospective registry of the WCD in which the risk of fatal arrhythmias during WCD use was assessed and the rate of LVEF improvement at the end of WCD use determined in patients at high risk for cardiac events [28]. Inappropriate shocks administered by WCDs were reported in only 0.5% of cases. At the end of WCD use (after coronary revascularization), 41% of patients experienced an improvement in LVEF and did not require ICD implantation 30 to 90 days after MI. These results provide evidence that WCDs can improve survival for patients with a high SCD risk while they wait for ICD implantation, as well as reduce unnecessary ICD implantation.

**Table 2:** Clinical studies of wearable cardioverter-defibrillators for patients who experienced myocardial infarctions

	WEARIT/BIROAD	WEARIT-II	VEST
Study design	Retrospective analysis	Prospective observational registry	Randomized trial
Number of patients	289	2000 (ICM group: *n* = 805)	2302 (WCD group: *n* = 1524)
Patients’ background	(1) NYHA III/IV HF with LVEF < 30% (2) IHD after PCI/CABG with LVEF < 30% or ventricular arrhythmia	ICM Non-ICM Congenital	MI with LVEF < 35%
Mean ± SD age, years	55 ± 12	62 ± 16	61 ± 12
Male sex, %	82	70	73
Mean ± SD baseline EF, %	23 ± 10	25 ± 10	28 ± 6
Mean (± SD) follow-up duration, days		90	84 ± 16
WCD use during follow-up, days	93 (mean)	90 (median)	58 (median)
Median hours per day wearing the WCD	NR	22.5	18.0
Arrhythmic death	NR	0.2%	WCD vs. Control: 1.6% vs. 2.4% RR (95% CI) 0.67 (0.37–1.21)
All-cause mortality	4.2%	0.2%	WCD vs. Control: 3.1% vs. 4.9% RR (95% CI) 0.64 (0.43–0.98)
Appropriate shocks, %	2.1	1.1	1.3
Inappropriate shocks, %	2.1	0.5	0.6

CABG, coronary artery bypass grafting; CI, confidence interval; EF, ejection fraction; HF, heart failure; ICM, ischemic cardiomyopathy; IHD, ischemic heart disease; LVEF, left ventricular ejection fraction; MI, myocardial infarction; NR, not reported; NYHA, New York Heart Association; PCI, percutaneous coronary intervention; RR, relative risk; SD, standard deviation; WCD, wearable cardioverter-defibrillator

**Fig. 3:**
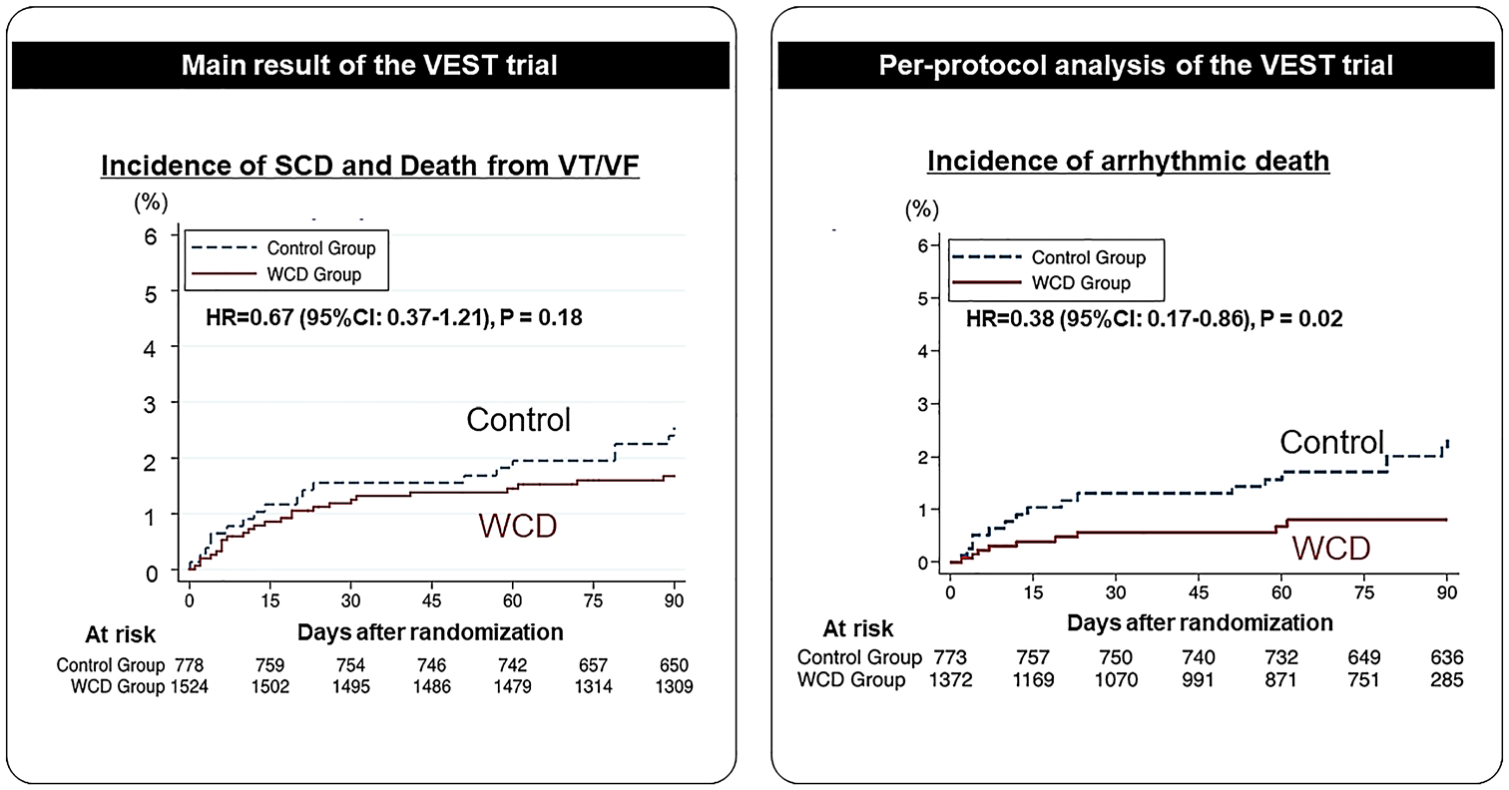
Kaplan–Meier analysis in the VEST trial. In intention-to-treat analysis, the incidence of sudden cardiac death and ventricular tachycardia/ventricular fibrillation was comparable between the wearable cardioverter-defibrillator (WCD) and control groups (**a**). Conversely, in the post hoc per-protocol analysis, the use of WCD was statistically significantly associated with a lower incidence of arrhythmic death compared with the control group (**b**)

The VEST trial was the only randomized controlled trial to date to evaluate the efficacy of WCDs in preventing arrhythmic and all-cause mortality in patients who experienced an MI and had a reduced LVEF [29]. Of 2302 patients who experienced an MI and had a LVEF < 35%, 1524 were randomized into a group receiving guideline-directed medical therapy with a WCD; the use of a WCD was not statistically significantly associated with a reduction of arrhythmic SCD (Fig. 3). Despite careful power calculations in the VEST trial, relatively low compliance in wearing the WCDs reduced the power of the study for demonstrating a survival benefit of WCDs. Although the investigators assumed a device-adherence rate of 70% in their trial, that goal was only met or exceeded in the first 2 weeks. Over time, the proportion of patients using the WCD waned to approximately 40%. Notably, the majority of deaths in the WCD arm (36 of 48 deaths) in the VEST trial occurred in patients who were not wearing the WCD. However, in as-treated and per-protocol analyses (subject to effect-cause bias and confounding by propensity to adhere), which assessed the impact of early cessation of WCD use on SCD, WCD use during the first 3 months after an MI was associated with a relative risk reduction compared to non-WCD use (Fig. 3) [30]. Eventually, although the main analysis of the VEST trial failed to show clinical benefit of WCD, the recent meta-analysis has demonstrated the clinical efficacy of WCD for terminating fatal ventricular arrhythmia in patients with elevated risk of SCD [31].

## Unresolved issues

Since the preventive effect of WCD is only conferred to patients when the device is worn, compliance with WCD use is a critical issue. The VEST trial demonstrated markedly lower daily wearing times compared to previous observational and prospective studies [20]. Indeed, by the end of the 90-day follow-up period in the VEST trial, the mean ± standard deviation daily wearing time decreased from 16.3 ± 9.8 h on day 1 to 8.3 ± 10.6 h [29]. Notably, in the VEST trial, the majority of mortalities among patients randomized to the WCD group occurred when participants were not wearing the WCD. It is desirable that medical professionals guide patients toward appropriate WCD use. The recent report demonstrated the novel ‘WCD training team’ approach for maintaining high WCD wearing compliance and this approach could be widely adapted for Japanese patients [32].

The cost of WCDs is one major barrier in the way of its widespread clinical application. The reimbursement for renting a WCD had not been covered completely by health insurance, resulting in a net expenditure by healthcare facilities. Therefore, the rental fee was revised in April 2020, resolving the imbalance. To receive this reimbursement, it is essential that clinicians assess patients’ condition monthly at the out-patient office.

Due to lack of awareness among interventional cardiologists, WCD is still under-recognized and has difficulty for routine use to prevent SCD in high-risk population during acute-phase. We hope this review will promote appropriate use of WCD in the modern PCI era.

## Conclusions

SCD after an MI remains a concern even in the era of primary PCI; therefore, optimizing the preventive strategy against SCD is essential, particularly in patients with a low LVEF following an MI. As conventional ICD implantation is not indicated during the early phases (within 3 months) after an MI, WCD could be used as a bridge to definitive therapy in such cases. However, the only randomized trial that has been conducted in this field could not demonstrate a statistically significant reduction of arrhythmic death in the post-MI population, which might have been due to the low patient adherence rate. In future, the importance of wearing the WCD should be clearly conveyed to participants; it will not only likely improve survival rates, but also the statistical power of trials. To promote patient compliance, medical providers, including interventional cardiologists, should be familiar with the appropriate use of the WCD; this should improve the prognosis in the post-MI population with left ventricular dysfunction.

## References

[1] Huikuri HV, Castellanos A, Myerburg RJ (2001). Sudden death due to cardiac arrhythmias. N Engl J Med.

[2] Chugh SS, Reinier K, Teodorescu C, Evanado A, Kehr E, Al Samara M (2008). Epidemiology of sudden cardiac death: clinical and research implications. Prog Cardiovasc Dis.

[3] Solomon SD, Zelenkofske S, McMurray JJV, Finn PV, Velazquez E, Ertl G (2005). Sudden death in patients with myocardial infarction and left ventricular dysfunction, heart failure, or both. N Engl J Med.

[4] Buxton AE, Lee KL, Fisher JD, Josephson ME, Prystowsky EN, Hafley G (1999). A randomized study of the prevention of sudden death in patients with coronary artery disease. Multicenter unsustained tachycardia trial investigators. N Engl J Med.

[5] Moss AJ, Zareba W, Hall WJ, Klein H, Wilber DJ, Cannom DS (2002). Prophylactic implantation of a defibrillator in patients with myocardial infarction and reduced ejection fraction. N Engl J Med.

[6] Steinbeck G, Andresen D, Seidl K, Brachmann J, Hoffmann E, Wojciechowski D (2009). Defibrillator implantation early after myocardial infarction. N Engl J Med.

[7] Hohnloser SH, Kuck KH, Dorian P, Roberts RS, Hampton JR, Hatala R (2004). Prophylactic use of an implantable cardioverter-defibrillator after acute myocardial infarction. N Engl J Med.

[8] Masuda M, Nakatani D, Hikoso S, Suna S, Usami M, Matsumoto S (2016). Clinical impact of ventricular tachycardia and/or fibrillation during the acute phase of acute myocardial infarction on in-hospital and 5-year mortality rates in the percutaneous coronary intervention era. Circ J.

[9] Berger CJ, Murabito JM, Evans JC, Anderson KM, Levy D (1992). Prognosis after first myocardial infarction. Comparison of Q-wave and non-Q-wave myocardial infarction in the Framingham Heart Study. JAMA.

[10] Shiga T, Kohro T, Yamasaki H, Aonuma K, Suzuki A, Ogawa H (2018). Body mass index and sudden cardiac death in Japanese patients after acute myocardial infarction: data from the JCAD study and HIJAMI-II registry. J Am Heart Assoc.

[11] Reitsma JB, Dalstra JA, Bonsel GJ, van der Meulen JH, Koster RW, Gunning-Schepers LJ (1999). Cardiovascular disease in the Netherlands, 1975 to 1995: decline in mortality, but increasing numbers of patients with chronic conditions. Heart.

[12] Boersma E, Mercado N, Poldermans D, Gardien M, Vos J, Simoons ML (2003). Acute myocardial infarction. Lancet.

[13] Ottervanger JP, Ramdat Misier ARR, Dambrink JHE, de Boer MJ, Hoorntje JC, Gosselink AT (2007). Mortality in patients with left ventricular ejection fraction ≤ 30% after primary percutaneous coronary intervention for ST-elevation myocardial infarction. Am J Cardiol.

[14] Mukharji J, Rude RE, Poole WK, Gustafson N, Thomas LJ, Strauss HW (1984). Risk factors for sudden death after acute myocardial infarction: two-year follow-up. Am J Cardiol.

[15] Wilber DJ, Zareba W, Hall WJ, Brown MW, Lin AC, Andrews ML (2004). Time dependence of mortality risk and defibrillator benefit after myocardial infarction. Circulation.

[16] Halkin A, Singh M, Nikolsky E, Grines CL, Tcheng JE, Garcia E (2005). Prediction of mortality after primary percutaneous coronary intervention for acute myocardial infarction: the CADILLAC risk score. J Am Coll Cardiol.

[17] Brooks GC, Lee BK, Rao R, Lin F, Morin DP, Zweibel SL (2016). PREDICT investigators. Predicting Persistent left ventricular dysfunction following myocardial infarction: The PREDICT Study. J Am Coll Cardiol.

[18] Klein HU, Meltendorf U, Reek S, Smid J, Kuss S, Cygankiewicz I (2010). Bridging a temporary high risk of sudden cardiac arrhythmic death. Experience with the wearable cardioverter defibrillator (WCD). Pacing Clin Electrophysiol.

[19] Dillon KA, Szymkiewicz SJ, Kaib TE (2010). Evaluation of the effectiveness of a wearable cardioverter defibrillator detection algorithm. J Electrocardiol.

[20] Chung MK, Szymkiewicz SJ, Shao M, Zishiri E, Niebauer MJ, Lindsay BD (2010). Aggregate national experience with the wearable cardioverter-defibrillator: event rates, compliance, and survival. J Am Coll Cardiol.

[21] Epstein AE, Abraham WT, Bianco NR, Kern KB, Mirro M, Rao SV (2013). Wearable cardioverter-defibrillator use in patients perceived to be at high risk early post-myocardial infarction. J Am Coll Cardiol.

[22] Feldman AM, Klein H, Tchou P, Murali S, Hall WJ, Mancini D (2004). Use of a wearable defibrillator in terminating tachyarrhythmias in patients at high risk for sudden death: results of the WEARIT/BIROAD. Pacing Clin Electrophysiol.

[23] JCS Joint Working Group (2016). Guidelines for non-pharmacotherapy of cardiac arrhythmias. Circ J.

[24] Priori SG, Blomström-Lundqvist C, Mazzanti A, Blom N, Borggrefe M, Camm J, ESC Scientific Document Group (2015). ESC Guideline for the management of patients with ventricular arrhythmias and the prevention of sudden cardia death: the task force for the management of patients with ventricular arrhythmias and the prevention of sudden cardiac death of the European Society of Cardiology (ESC). endorsed by: association for European pediatric and congenital cardiology (APEC). Eur Heart J.

[25] Piccini JP, Allen LA, Kudenchuk PJ, Page RL, Patel MR, Turakhia MP, American Heart Association Electrocardiography and Arrhythmias Committee of the Council on Clinical Cardiology and Council on Cardiovascular and Stroke Nursing (2016). Wearable cardioverter-defibrillator therapy for the prevention of sudden cardiac death: a science advisory from the American Heart Association. Circulation.

[26] Al-Khatib SM, Stevenson WG, Ackerman MJ, Bryant WJ, Callans DJ, Curtis AB (2018). AHA/ACC/HRS guidelines for management of patients with ventricular arrhythmias and the prevention of sudden cardiac death: a report of the American college of cardiology/American Heart association task force on clinical practice guidelines and the heart rhythm society. J Am Coll Cardiol.

[27] Zishiri ET, Williams S, Cronin EM, Blackstone EH, Ellis SG, Roselli EE (2013). Early risk of mortality after coronary artery revascularization in patients with left ventricular dysfunction and potential role of the wearable cardioverter defibrillator. Circ Arrhythm Electrophysiol.

[28] Kutyifa V, Moss AJ, Klein H, Biton Y, McNitt S, MacKecknie B (2015). Use of the wearable cardioverter defibrillator in high-risk cardiac patients: data from the Prospective Registry of Patients Using the Wearable cardioverter Defibrillator (WEARIT-II Registry). Circulation.

[29] Olgin JE, Pletcher MJ, Vittinghoff E, Wranicz J, Malik R, Morin DP (2018). Wearable cardioverter–defibrillator after myocardial infarction. N Engl J Med.

[30] Olgin JE, Lee BK, Vittinghoff E, Morin DP, Zweibel S, Rashba E (2020). Impact of wearable cardioverter-defibrillator compliance on outcomes in the VEST trial: as-treated and per-protocol analyses. J Cardiovasc Electrophysiol.

[31] Nguyen E, Weeda ER, Kohn CG, D’Souza BA, Russo AM, Noreika S, Coleman CI (2018). Wearable Cardioverter-defibrillators for the Prevention of Sudden Cardiac Death: a meta-analysis. J Innov Card Rhythm Manag.

[32] Horiguchi A, Kishihara J, Niwano S, Saito D, Matsuura G, Sato T (2020). Wearable cardioverter defibrillator—initial experience in the outpatient setting in Japan. Circ Rep.

